# Analyse des facteurs histo-pronostiques du cancer du rectum non métastatique dans une série ouest Algérienne de 58 cas au CHU-Tlemcen

**DOI:** 10.11604/pamj.2016.24.5.8580

**Published:** 2016-05-03

**Authors:** Smain Nabil Mesli, Derbali Regagba, Anisse Tidjane, Mokhtar Benkalfat, Chakib Abi-Ayad

**Affiliations:** 1 Service de Chirurgie Viscérale «A», Centre Hospitalier Universitaire Tlemcen, Laboratoire de Recherche N°39, Faculté de Médecine, Tlemcen, Algérie; 2 Service d'Epidémiologie Centre Hospitalier Universitaire, Oran, Algérie; 3 Service de Chirurgie Hépatobiliaire et Greffe du Foie EHU- 1er Novembre 1954, Oran, Algérie; 4 Laboratoire de Chirurgie Expérimentale N°39, Faculté de Médecine, Tlemcen, Algérie

**Keywords:** Adénocarcinome rectale, survie, récidive, Rectal adenocarcinoma, survival, recurrence

## Abstract

**Introduction:**

L'objectif de notre travail est d'analyser les facteurs histo-pronostiques des cancers du rectum non métastatique opérés au service de chirurgie «A» de Tlemcen à ouest Algérien durant une période de six ans.

**Méthodes:**

Etude rétrospective de 58 patients qui avait un adénocarcinome rectal. Le critère de jugement était la survie. Les paramètres étudiés, le sexe, l’âge, stade tumoral, et les récidives tumorales.

**Résultats:**

L’âge moyen était de 58 ans. Avec 52% d'hommes contre 48% femmes avec sex-ratio (1,08). Le siège tumoral était: moyen rectum avec 41,37%, 34,48% au bas rectum et dans 24,13% au haut rectum. La classification TNM avec 17,65% au stade I, 18,61% au stade II, 53, 44% au stade III et 7,84% au stade IV. La survie médiane globale était de 40 mois ±2,937 mois. La survie en fonction du stade tumoral, le stade III et IV avait un faible taux de survie (19%) a 3 ans contre le stade I, II avait un taux de survie de (75%) (P = 0,000) (IC 95%). Les patients avec récidives tumorales avaient un taux de survie faible à 3 ans par rapport à ceux n'ayant pas eu de récidive (30,85% Vs 64,30% P = 0,043).

**Conclusion:**

Dans cette série, l’étude uni varié des différents facteurs pronostiques conditionnant la survie n'a permis de retenir que trois facteurs influençant la survie, à savoir la taille tumorale, le stade, et les récidives tumorales. En analyse multi variée en utilisant le modèle Cox un seul facteur été retenu la récidive tumorale.

## Abstract (en)

**Introduction:**

The aim of our study was to analyze histoprognostic factors in patients with non-metastatic rectal cancer operated at the division of surgery “A” in Tlemcen, west Algeria, over a period of six years.

**Methods:**

Retrospective study of 58 patients with rectal adenocarcinoma. Evaluation criterion was survival. Parameters studied were sex, age, tumor stage, tumor recurrence.

**Results:**

The average age was 58 years, 52% of men and 48% of women, with sex-ratio (1,08). Tumor seat was: middle rectum 41.37%, lower rectum 34.48% and upper rectum 24.13%. Concerning TNM clinical staging, patients were classified as stage I (17.65%), stage II (18.61%), stage III (53.44%) and stage IV (7.84%). Median overall survival was 40 months ±2,937 months. Survival based on tumor staging: stage III and IV had a lower 3 years survival rate (19%) versus stage I, II which had a survival rate of 75% (P = 0.000) (95%). Patients with tumor recurrences had a lower 3 years survival rate compared to those who had no tumoral recurrences (30.85% vs 64.30% P = 0.043).

**Conclusion:**

In this series, univariate analysis of prognostic factors affecting survival allowed to retain only three factors influencing survival: tumor size, stage and tumor recurrences. In multivariate analysis using Cox's model only one factor was retained: tumor recurrence.

## Introduction

Le cancer du rectum (CR) est souvent intégré dans les îlots des cancers colorectaux (CCR). Il est de plus en plus fréquent et pose un réel problème de diagnostic et de prise en charge dans les pays en développement [1]. En Algérie, son incidence annuelle est de 31,8 cas pour 100 000 habitants, soit une moyenne annuelle de 1500 cas incident. Le CR se situe au 2^émè^ rang des cancers digestifs [2]. Il représente la deuxième cause de mortalité dans les pays développés [1–3]. Les progrès réalisés en matière de diagnostic et thérapeutique (Traitement néo-adjuvant) ont amélioré la survie qui ne dépasse pas 50% à 5 ans [4]. Reconnaître les facteurs pronostics du CR conditionne la survie à long terme. Déterminer ces facteurs est d'une importance capitale permettant ainsi d'orienter les patients vers un Protocol thérapeutique plus adéquat avec un calendrier de surveillance mieux adapté. L'objectif de notre travail est d'analyser les facteurs histo-pronostiques des cas de cancer du rectum pris en charge durant une période de six ans au service de chirurgie «A» du centre hospitalo-universitaire de Tlemcen à ouest Algérien.

## Méthodes

Répondre à la problématique sus énoncé, une étude pronostic mono centrique a été menée, portant sur les dossiers de malades admis et opérés pour CR au service de chirurgie viscérale «A» au centre hospitalo-universitaire de Tlemcen, sur une période de 6 ans allant de Janvier 2009 à Mai 2015. Nous avons inclus dans cette étude tout patient présentant un adénocarcinome prouvé histologiquement et dont le siège de la tumeur était situé entre 0 et 12 cm de la marge anale. Était exclus l’étude, tout adénocarcinome rectal associé à des métastases à distance (hépatiques, pulmonaires, péritonéales), une tumeur située au delà de 12 cm le la marge anale et ceux de la jonction récto-sigmoïdienne, ainsi que toute tumeur rectal non glandulaire, un traitement non curatif, cancer opéré en urgence et enfin tout dossier incomplet. Cinquante-huit patients qui réunissaient les critères de l’étude étaient retenus, pour lesquels une courbe de survie a été réalisée. Les paramètres étudiés étaient, le sexe, l’âge, le siège tumoral, le degrés de différenciation de l'adénocarcinome, le dosage des anti gènes carcino-embryonnaires (ACE), les traitements associés a celui de la chirurgie (néo-adjuvant et adjuvant), le type de traitement chirurgical, nombres de ganglions envahis, stade tumoral, et enfin l'existence ou non d'une récidive tumorale. Pour ce qui est de l'analyse statistique, on procède d'abord à une description de la population de l’étude on exprimons par des pourcentages pour des données qualitatives et sous forme de moyenne ± écart type pour les données quantitatives, puis une analyse de survie globale et en fonction des facteurs pronostics on utilisera la méthode de Kaplan Meier tout on estimons la moyenne et la médiane de survie avec des comparaisons de la survie on fonction des facteurs pronostic par le test de Long-Rang (p < 0,05).

Les facteurs pronostics ayant un seuil de signification statistique < ou = 3% étaient introduites dans un modèle de régression Cox pour l'analyse multi-variée.

## Résultats

Sur l'ensemble des 86 dossiers d'adénocarcinome du rectum, nous avons colligé 58 patients qui présentaient un cancer du rectum prouvé histologiquement et dont les dossiers étaient complets. L’âge moyen des patients étaient de 58 ans ± 11,6 avec des extrêmes «30-84ans». Il s'agissait de 52% d'hommes (n = 30) et de 48% de femmes (n = 28) avec sex-ratio (1,08). L'examen endoscopique montrait que la tumeur était située au moyen rectum dans 41,37% (n = 24), 34,48% (n = 20) au bas rectum et dans 24,13% (n = 14) au niveau du haut rectum. Sur le plan histologique la biopsie avait montré que l'adénocarcinome liberkunien était bien différencié dans 75,86% (n = 44), moyennement différencié dans 20,68% (n = 12) et dans 3,44% (n = 2) peu différencié. L'antigène carcino-embryonnaire (ACE) été dosé dans 48,77% (n = 28) et révélait un taux supérieur à 5ng/ml chez 28,57% (n = 8) patients. Sur le plan thérapeutique, un traitement néo-adjuvant par radio-chimiothérapie (RCC) était pratiqué chez 18,95% (n = 11) et une radiothérapie seul cycle court pour 6,90% (n = 4). Une exérèse chirurgicale de type amputation abdomino pelvienne (AAP) était réalisée chez 22,41% des cas (n = 13), alors qu'une chirurgie conservatrice de l'appareil sphinctérien était possible dans 77,58% des cas (n = 45). Les suites postopératoires étaient marquées par une mortalité de l'ordre de 8,62% (n = 5), et une morbidité de 24,15%(n = 14). La durée de séjour hospitalier était de 17, 90 jours ±6,24 avec des extrêmes de «6-32 jours». L'examen anatomopathologique sur pièce opératoire avait précisé que la taille moyenne tumorale était de 4,42 cm ±1,98 avec des extrêmes «1-8cm» et que la taille tumorale était supérieure à 5 cm dans 15,70% (n = 10) et inférieure à 5 cm dans 84,30% (n = 48). En analysant l'envahissement ganglionnaire, le curage avait permis de prélever en moyenne 10,77 ganglions ±5,513 avec des extrêmes de «00-33». Étaient envahis en moyenne 2,25 ganglions ±2,928 avec des extrêmes de «00-17». L’étude histologique avait permis de classer les patients selon la classification TNM avec 17,65% des patients au stade I (n = 10),18,61% au stade II (n = 11), 53,44% au stade III(n = 33) et 7,84% au stade IV (n = 4). En postopératoire vingt deux patients (41,50%) avait bénéficié d'une radio-chimiothérapie, vingt neuf patients (54,71%), d'une chimiothérapie systémique et deux patients d'une radiothérapie seule (3,77%).

**Figure 1:**
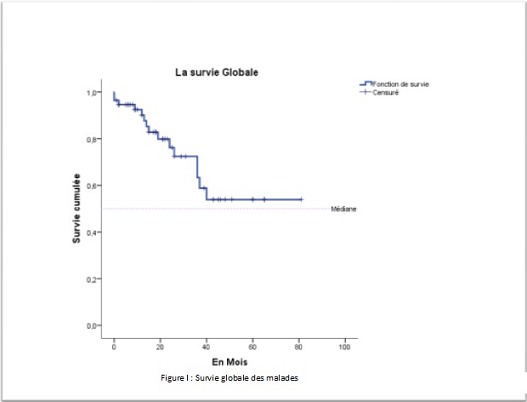
Analyse de la survie globale des malades opères pour cancer du rectum non métastatique

**Figure 2:**
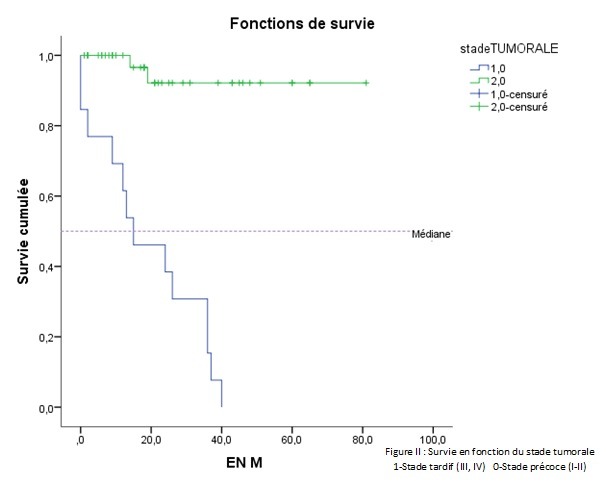
Analyse de la survie en fonction du stade de la maladie tumorale, 1-Stade tardif (III, IV) 0-Stade précoce (I-II)

**Figure 3:**
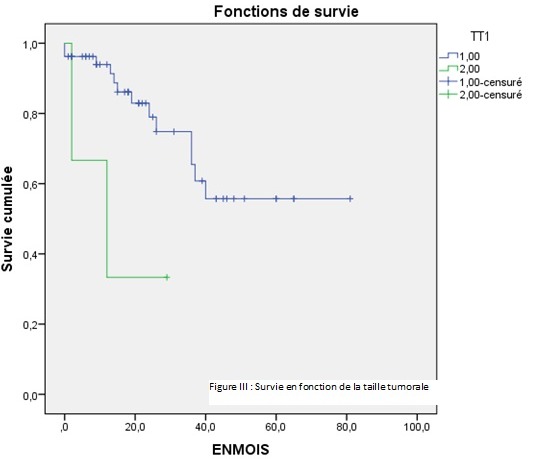
Analyse de la survie en fonction de la taille tumorale en centimètres

**Figure 4:**
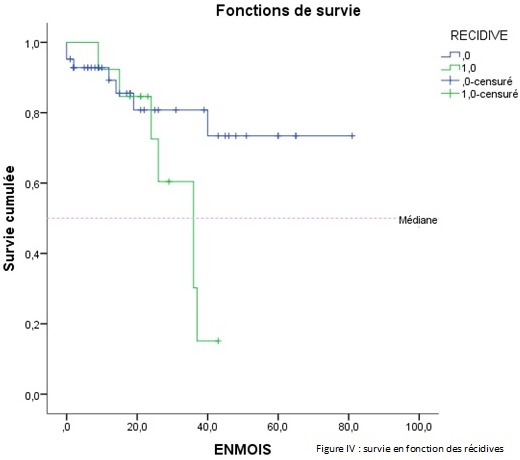
Analyse de la Survie en fonction des récidives tumorale

Par ailleurs, dix patients (18,86%) avaient présenté une récidive et dont le délai moyen était de 18,90±9,53 mois avec des extrêmes de« 6-36 mois» (Tableau 1). La survie médiane globale était de 40 mois ±2,937 mois (Figure 1). L'analyse univariée de la survie en fonction du sexe ne retrouvait pas de différence significative P = 0,661. De même en comparant la survie en fonction de l’âge, entre un groupe de patients moins de 50 ans et un groupe âgé plus de 50 ans. Le siège de la tumeur n'avait aucune différence significative sur la survie entre le haut, moyen et bas rectum. Selon le geste pratiqué, celui-ci ne montrait pas une différence significative entre un geste ne conservant pas l'appareil sphinctérien (AAP) et un geste conservant l'appareil sphinctérien (RA) (Tableau 2). La survie en fonction d'un traitement néo adjuvant n'avait pas de différence significative. En analysant la survie en fonction du stade tumoral, le stade III et IV avait un faible taux de survie (19%) a 3 ans tandis que le stade I, II avait un taux de survie de (75%) à 3 ans. (P = 0,000) (IC 95%) (Figure 2). La survie à 3 ans en fonction de la taille tumorale était significativement différente, lorsque la taille tumorale était supérieure à 5 cm. La survie était faible par rapport à une taille inférieur à 5 cm (P = 0,021) (Figure 3). Les patients ayant présenté des récidives tumorales avait un taux de survie faible à 3 ans par rapport à ceux n'ayant pas eu de récidives tumorales (30,85% contre 64,30% P = 0,043) (Figure 4). D'autres facteurs ont été analysés, tel que l'existence d'un traitement adjuvant, l'envahissement ganglionnaire et le taux de l'ACE. Mais il n'avait aucune influence sur la survie dans notre étude. L'analyse univariée avait permis l'identification de trois variables significatives (taille, stade tumorale et récidive). Ces derniers étaient inclus dans un modèle de Cox et un seul facteur déterminant à savoir la récidive tumorale qui sortait et avait une influence significative sur la survie à trois ans.

**Tableau 1:** Caractéristiques des patients dans notre série selon les variables étudiés

Patients	Nombre de malade
Variables	Résultats
Age	58 ans ± 11,6
Sexe	52% d'hommes sex-ratio (1,08)
Siege tumorale (Haut, moyen, bas)	(41,37%, 34,48%, 24,13%)
^a^Taux ACE > 5ng/ml	28,57%(n = 8)
Type histologique **:** -bien différentié	75,86%
-Moyennement différentié	20,68%
-peu différentie	3,44%
^b^RCC	18,95%
radio thérapie seule	6,90%
APP / RA	(22,41% - 77,58%)
Morbidité	24,14%
Mortalité	8,62%
Récidive	18,86%

a 28 patients ont eu le dosage

b radio chimiothérapie préopératoire

**Tableau 2:** Analyse univariée des facteurs influençant la des malades opérés pour cancer du rectum non métastatique dans notre série

Variables significatives P < 0,05	Variables non Significatives 0,05 < p <0,10	Valeur non significatives P > 0,10
-Taille tumorale P = 0,0223	-Geste opératoire RA/APP	-Age
-Stade tumorale	-Siège tumorale	-Sexe
-Précoce / tardive P = 0,0000	-Traitement néo-adjuvant	-Tare
-Récidive P = 0,0000	-Traitement adjuvant	

## Discussion

L'incidence du CR est plus élevée dans les pays du nord qu'en Afrique [5]. Comme très peu de régions en Afrique sont couvertes par un registre, en Algérie l'incidence du CR reste sous-estimée et difficile à préciser. Et d'après les dernier données épidémiologiques publiées en 2009 [2], le CR en Algérie occupe le 4^ème^ rang parmi les cancers chez l'homme et le 5^ème^ chez la femme. L’âge moyen de nos patients était de 58 ans comparable à celui rapporté aux autres séries Africaines Nigérienne et Béninoise [6, 7] où l’âge moyen variait entre 46,7 à 51,2 ans. Ainsi, le CR apparaît à un âge relativement plus bas chez les africains que chez les occidentaux où le pic de fréquence se situe entre 60 et 70 ans [5]. Nos patients semblent plus jeunes en raison de la jeunesse de notre population. Dans notre série le sex-ratio était de 1,08 identique à celui qui était retrouvé dans la littérature [8, 9]. L’étude des facteurs pronostiques permet au clinicien de sélectionner les patients pour un traitement donné. Si le principal facteur reste le stade évolutif de la tumeur au moment du diagnostic [10], il est important de déterminer d'autres facteurs pronostiques qui conditionnent la survie. Parmi ces facteurs pronostiques cliniques étudiés dans la littérature: l’âge, ce dernier reste un facteur discutable. Six études sur quinze qui évaluaient ce facteur avaient conclu que la survenue d'un CR chez le sujet jeune était de mauvais pronostic [11]. Dans notre étude nous n'avions pas trouvé de différence significative en termes de survie en fonction des tranches d’âge de nos patients. La prédominance masculine était dominante dans notre série. Trois études multi variées avaient affirmé que la survie à long terme était meilleure chez la femme par rapport à l'homme [12–15]. Cette constatation n’était pas identifiée dans notre série puisque nous n'avons pas trouvé de différence significative de survie entre les deux sexes. Selon la topographie et en comparant la survie en fonction du siège tumoral, une étude de Jatzco [16] qui étudiait l'influence du siège tumoral sur la survie et ces constatations avaient conclus qu'il n'y avait pas d'influence. Il en est de même dans notre série où il n'y avait pas de différence significative selon le siège tumoral (P = 0,123).

Sur le plan biologique, parmi les marqueurs tumoraux, l'ACE est le marqueur tumoral le plus utilisé en pathologie colorectale. Toute élévation de ce marqueur en pré-opératoire, était un facteur de mauvais pronostic dans plusieurs études publiées [17, 18, 10]. Dans notre série, l’élévation du taux sérique de l'ACE n’était pas un facteur influençant la survie. En ce qui concerne les facteurs thérapeutiques étudiés, depuis la fin des années 90, l'association de la chimiothérapie à la radiothérapie a encore amélioré le pronostic carcinologique et fonctionnel du CR. Une méta-analyse réalisée en 2013 [19] qui comparait la radio-chimiothérapie néo-adjuvante versus chirurgie seule, avait prouvé qu'il n'y avait pas de différence significative sur la survie globale à long terme. Dans notre étude, il n'y avait pas de différance significative (p = 0,576) entre un traitement néo-adjuvant chirurgie versus chirurgie seule. Mais ceci reste à prendre avec précaution car l’échantillon de notre série était faible. En ce qui concerne le traitement chirurgical, deux études prospectives réalisées chez 2136 et 1219 malades [20, 21] avaient comparé les différentes techniques chirurgicales à savoir les amputations abdomino-périnéales et une résection antérieure pour la tumeur du moyen et bas rectum. Ces études n'avaient pas trouvé de différence significative sur la survie globale. Ce qui a été retrouvé dans notre étude. En dehors des facteurs pronostiques cliniques, biologiques et thérapeutiques, d'autres facteurs d'importance capitale ont été étudiés par différentes études. C'est l’étude anatomopathologique qui a analysé l'aspect macroscopique et microscopique de la tumeur. Parmi les facteurs analysés macroscopiquement, c'est l'influence de la taille tumorale sur la survie, qui reste controversée dans la littérature. Park JY et He WJ [22, 23] rapportait dans leurs analyses multi-variées que la taille de la tumeur n’était pas un facteur pronostic influençant la survie. Par contre, d'autres études ont prouvé le contraire tel l’étude de Xu FY qui avait trouvé que lorsque la taille supérieure à 6 cm était de mauvais pronostic [24]. Dans notre analyse, la taille tumorale était un facteur influençant sur la survie globale de façon significative (P = 0,023). En ce qui concerne l'influence de l'envahissement ganglionnaire du CR sur la survie, des études ont confirmés cette influence sur la survie [25, 26]. Pour notre part l'envahissement ganglionnaire et le nombre de ganglions n’étaient pas un facteur influençant. En comparant la survie des différents stades tumoraux dans notre série, les stades III et IV (19% a trois ans) avaient un taux de survie plus faible, tandis que les stades I et II avaient meilleur taux de survie (79% à trois ans). Ces mêmes résultats sont retrouvés dans 2 études multi variées comparant les stades tumoraux [15–27]. Dans une étude analytique Tunisienne réalisée en 2006 [10], qui avait démontré que les patients présentant une récidive avaient un taux de survie plus faible. Cette conclusion a été retrouvé dans notre analyse, puisqu'il n'y'avait une différence significative entre les deux groupes (P < 10^3^)

## Conclusion

Dans cette série, l’étude univarié des différents facteurs pronostiques conditionnant la survie n'a permis de retenir que trois facteurs influençant la survie, a savoir la taille tumorale, le stade, et les récidives tumorales. En analyse multi variée en utilisant le modèle Cox un seul facteur été retenu c'est la récidive tumorale

### Etat des connaissances actuelles sur le sujet

- La survie est influencée par deux facteurs: la taille tumorale; la récidive tumorale.

### Contribution de notre étude à la connaissance

- Les facteurs suivant n'influencent pas la survie: le type de chirurgie, le siège de la tumeur, l'association ou non à un traitement néo-adjuvant;
- On confirme que la taille tumorale et la récidive ont une influence sur la survie.
